# *Staphylococcus aureus* Colonization Modulates Tic Expression and the Host Immune Response in a Girl with Tourette Syndrome

**DOI:** 10.3389/fpsyt.2016.00031

**Published:** 2016-03-14

**Authors:** Costantino Eftimiadi, Gemma Eftimiadi, Piergiuseppe Vinai

**Affiliations:** ^1^Medicina Generale Convenzionata – ASL CN1, Carrù, Italy; ^2^GNOSIS Research Group NPO, Magliano Alpi, Italy

**Keywords:** *Staphylococcus aureus*, *Streptococcus pyogenes*, Tourette syndrome, dopamine receptor autoantibodies, ASO, PANDAS, nasal carriage

## Abstract

A 9-year-old girl with Tourette syndrome (TS) and increased antibody levels against *Streptococcus pyogenes* was monitored longitudinally for the presence of nasopharyngeal bacteria, specific antibody titers, and autoimmunity directed against brain antigens. Microbiological monitoring indicated that the child was an intermittent *Staphylococcus aureus* nasopharyngeal carrier. Clinical improvements in motor tic frequency and severity were observed during the *S. aureus* colonization phase and were temporally correlated with the downregulation of anti-streptococcal and anti-D1/D2 dopamine receptor antibody production. After decolonization, clinical conditions reverted to the poor scores previously observed, suggesting a possible role of the immune response in bacterial clearance as a trigger of symptom recrudescence. These findings imply that a cause–effect relationship exists between *S. aureus* colonization and tic improvement, as well as between bacterial decolonization and tic exacerbation. Understanding the impact of *S. aureus* on the host adaptive immune response and the function of autoantibodies in the pathogenesis of TS may alter approaches for managing autoimmune neuropsychiatric and tic disorders.

## Introduction

Children with Tourette syndrome (TS) might be more prone to group A streptococcal (GAS) infections and could develop higher antibody titers against the pathogen than healthy controls (1). This signifies the existence of an underlying immunological disorder (2, 3). However, GAS infections are unlikely to exert a major effect on the severity of neuropsychiatric symptoms years after symptom onset (1). Nevertheless, other studies support a role of GAS infections and basal ganglia autoimmunity in a subgroup of patients with TS and suggest a similarity between patients with Sydenham’s chorea and some patients with either TS (4) or chronic recurrent episodic acute exacerbations of tic and obsessive–compulsive indications (5), according to the pathogenesis of pediatric autoimmune neuropsychiatric disorders associated with streptococcal infection (PANDAS) described by Swedo (6). Sydenham’s chorea was the first neuropsychiatric condition in which antibodies produced in response to GAS infections were found to cross-react with extracellular and intracellular targets in the basal ganglia, resulting in a disease state (7–9). Despite this finding, no conclusive data are available (10, 11), and the hypothesis that PANDAS and TS could be secondary to pathogenic autoantibodies is controversial (12, 13). Moreover, a consensus regarding the possible contribution of GAS infections to the etiology of tic disorders, especially TS, does not exist. Additionally, other pathogens can also induce postinfectious Tourette-like syndromes, including *Mycoplasma pneumonia* (14), *Borrelia burgdorferi* (15), and assorted viruses (16–18).

## Case Description

This study longitudinally investigated the possible associations between bacterial pathogens in nasopharyngeal and tonsil swabs, serological immune responses, and tic severity in an adolescent girl with TS, who first came to our attention in June 2012, when she was 9 years old. The patient exhibited a strong recrudescence of motor tics in the presence of high titers of anti-streptolysin O (ASO) antibodies (472 IU/mL; positive = >200 IU/mL).

A historical record released by the children’s neuropsychiatric public department of Azienda Sanitaria Locale Cuneo 1 (ASL CN1) indicated that the tic disorders began when the patient was 7 years old. Learning disabilities at school, borderline intellectual functioning, and phobic anxiety disorder were also present at that time. Routine laboratory analyses were normal, and there was no record of any previous GAS infection.

Informed parental consent was obtained to enroll the child in a clinical and laboratory survey, according to the principles outlined in the Declaration of Helsinki (1964). The aims of this study, negligible risks associated with the investigation, and the prospective benefits to the community for better scientific knowledge of the pathogenesis of TS and other tic disorders were discussed in detail. The protocol included physical examinations, interviews, completion of questionnaires, and periodic drawings of small amounts of blood to measure ASO, anti-streptococcal deoxyribonuclease B (DNase B), and anti-staphylolysin antibody (ASTA) titers, as well as white blood cell (WBC) counts and the erythrocyte sedimentation rate (ESR). Duplicate throat and nasal swabs were obtained for the microbiological assays and were analyzed on the same day by two different laboratories: the Centro Diagnostico Cernaia (Cuneo, Italy) and the Microbiology Department of ASL CN1 (Mondovì, Cuneo, Italy). An analysis for autoimmunity against brain antigens was conducted three times during the final 18 months of the study by the Wieslab AB Medical Laboratory (Malmo, Sweden), utilizing a test panel (Cunningham Panel) originally developed by Moleculera Labs (Oklahoma City, OK, USA). The assay comprised measurements of antibody titers against dopamine D1 and D2 receptors, lysoganglioside-GM1, and beta-tubulin, in addition to antibodies that induce the activation of calcium/calmodulin-dependent protein kinase type II (CaM kinase II) by binding to receptors on neural cell lines (7–9). Tic severity was monitored according to the Yale global tic severity scale (YGTSS; subscale 0–50).

During her first visit in June 2012, the patient’s tic severity score was 25, essentially indicating the presence of motor tics alone, and her ASO titer was elevated (472 IU/mL; positive = >200 IU/mL). Her ASO titers remained high (between 350 and 400 IU/mL) for almost 3 years. Additionally, her anti-DNase B titers were always in the upper range (between 360 and 340 IU/mL; positive = >200 IU/mL). The results of enzyme-linked immunosorbent assay (ELISA) analyses for ongoing autoimmunity against brain antigens are shown in Table 1. Four autoimmunity tests out of a panel of five were significantly positive based on the reference values of the test panel manufacturer (Moleculera Labs) (Table 1, observation period A: before *Staphylococcus aureus* colonization). Meanwhile, ELISA tests for antinuclear antibodies were negative throughout the study (data not shown).

**Table 1:** **Autoimmunity tests against brain antigens, anti-staphylolysin antibodies (ASTA), WBCs, and erythrocyte sedimentation rate (ESR), before (A), after (B), and during (C) *Staphylococcus aureus* colonization**.

Dopamine RD1 (titer)^a^			Dopamine RD2 (titer)^a^			Lysoganglioside (titer)^a^			Tubulin (titer)^a^			CaM kinase II (% of baseline)^b^			ASTA (IU/mL)			WBCs (×10^3^ μL)			ESR (mm/h)		
A	B	C	A	B	C	A	B	C	A	B	C	A	B	C	A	B	C	A	B	C	A	B	C
**8000**	Neg	Neg	**16000**	Neg	Neg	Neg	Neg	Neg	**2000**	**4000**	**4000**	**156**	**190**	**160**	1	**8**	2	13.20	**13.70**	7.03	8	**25**	11
Positive >2000			Positive >8000			Positive >320			Positive >1000			Positive >130			Positive >2			Positive >13.5			Positive >15		

**Figure 1:**
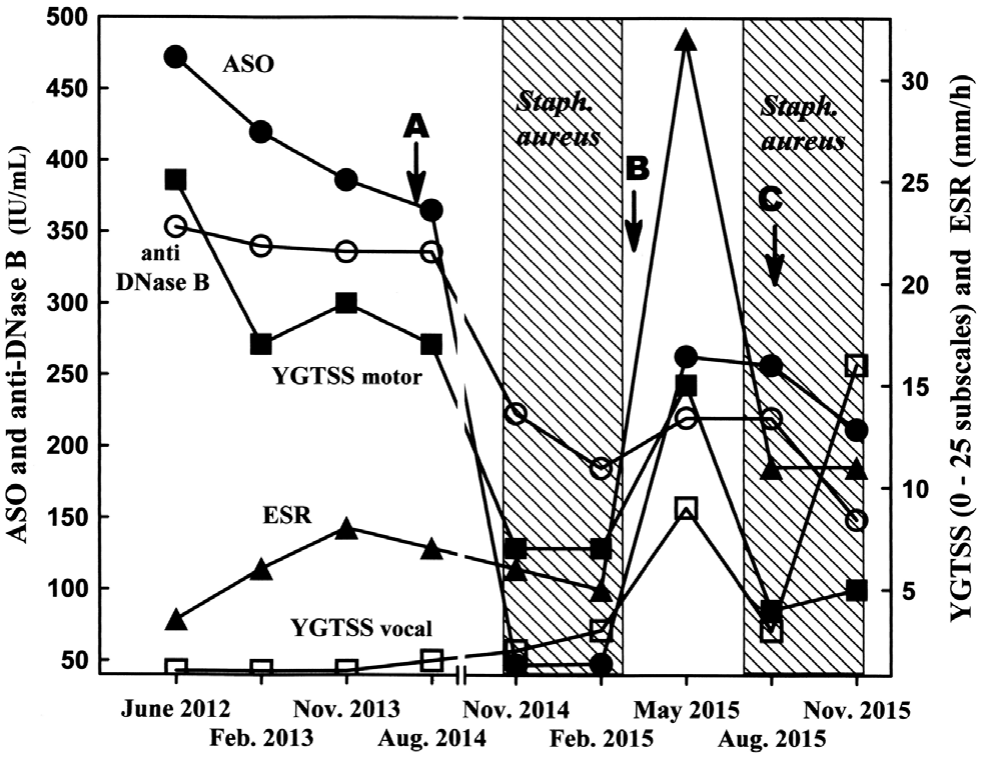
**Clinical and laboratory records**. Arrows indicate the times of blood sampling for autoimmunity testing. ASO, anti-streptolysin O antibody (filled circles); anti-DNase B, anti-deoxyribonuclease B antibody (open circles); ESR, erythrocyte sedimentation rate (filled triangles); YGTSS motor, Yale global tic severity score motor subscale (filled squares); YGTSS vocal, Yale global tic severity score vocal subscale (open squares).

*Blood samples for observation periods A, B, and C were drawn at the time-points indicated in Figure 1*.

*The Bold fonts highlight the positive experimental values*.

*^a^Titers are given as the minimum serum dilution required in ELISA assays to achieve a positive reading*.

*^b^Compared with standard values of normal controls in a test panel provided by Moleculera Labs, Inc. (Oklahoma City, OK, USA)*.

Combined pharmacological treatment with pimozide (4 mg/day) and sertraline, a selective serotonin reuptake inhibitor (50 mg/day), effectively controlled eye, head and shoulder movements, and comorbidities, both in frequency and severity. However, the complete elimination of trunk and abdominal movements, with tensing of the abdomen and urinary incontinence, was only slowly achieved. The patient’s clinical situation remained stable over the following 2 years, permitting a reduction in the daily doses of pimozide and sertraline to 1 and 25 mg/day, respectively. In late 2014, an unusual improvement in motor tics was initially recorded by the family. Subsequent laboratory tests showed a significant decrease in the ASO titer to normal levels, along with a decrease in the anti-DNase B titer to reference values (i.e., below 200 IU/mL) (Figure 1). Moreover, antibody titers against dopamine D1 and D2 receptors yielded negative values (Table 1, observation period B: after *S. aureus* colonization) in the absence of any antibiotic treatment. Microbiological analyses of nasal and throat swabs revealed, for the first time since beginning the survey, the presence of *S. aureus*. Antibiotic susceptibility testing indicated that this bacterial strain was sensitive to 11 antibiotics out of a panel of 12, with the only resistance being to benzylpenicillin. The patient’s YGTSS score dropped to seven, the lowest value recorded. Starting in November 2014, a clinical and laboratory survey was scheduled every 3 months, as well as once-monthly microbiological assays for the patient’s *S. aureus* carrier state.

A few months later, the scenario was completely reversed (Figure 1). *S. aureus* was no longer detected, and tic frequency and intensity rapidly returned to their previous poor scores with a combined symptomatology of motor and vocal tics. Parallel blood tests indicated a slight increase in WBC count (Table 1, observation period B), an ASO titer that increased from 47 to 264 IU/mL (Figure 1), an increase in the ESR (Figure 1), and a significantly positive ASTA antibody titer (Table 1, observation period B). These data indicate a shift from an anti-inflammatory state (colonization) to a pro-inflammatory condition, coincident with staphylococcal clearance by the host. The staphylococcal colonization was indeed temporary, or rather recurrent, a situation historically occurring in ~30% of the normal population (19). Four months later, a *S. aureus* strain with the same pattern of antibiotic susceptibility as that described above (and possibly the same strain) was again recovered from nostril and tonsil swabs; a significant clinical improvement was also observed (Figure 1). The clinical improvement was in fact so impressive that all pharmacological treatments were suspended. Autoantibodies against dopamine receptors were negative (Table 1, observation period C: during *S. aureus* colonization), as noted after the first colonization phase (Table 1, observation period B). However, anti-streptococcal antibody titers failed to decrease as rapidly as during the first colonization phase. Two months later, close to the end of the second colonization phase, vocal tic frequency and severity (grunts) increased to values never recorded before, with only a slight increase in motor tics (Figure 1). Microbiological analysis for the presence of GAS infections in pharyngo–tonsillar and nasal swabs was always negative.

## Background

To our knowledge, this is the first investigation of the role of *S. aureus* nasal carriage in a patient with a tic disorder. *S. aureus* is a bacterial pathogen equipped with a tremendous variety of virulence factors (20), and its phagocytosis by neutrophils requires a much higher expenditure of energy than that required for saprophytic strains (21). *S. aureus* is permanently present in about 20% of the general population, while ~30% transiently carry the pathogen and ~50% are not carriers (19). Nevertheless, host immunological response patterns indicate only two categories: carriers and non-carriers (19).

The nostril affords the main ecological niche where *S. aureus* resides in human beings, but the determinants of carrier state are not fully understood. Different hosts (22) and bacterial virulence factors (23–27) contribute to staphylococcal colonization and could potentially influence the immunological response and the carrier status. Competition for the same biological niche and/or direct antagonistic effects by different bacterial species might in principle interfere with *S. aureus* colonization, as experiments with *Staphylococcus epidermidis* have demonstrated (28, 29). Existing models suggest that *S. aureus* colonization modulates host immune responses, inducing tolerance and suppression of pro-inflammatory reactions (22). Increased production of interleukin (IL)-10 by monocyte-derived macrophages of the nasal submucosa (30), as well as bacterial superantigen induction of regulatory T cells (Tregs), is reportedly involved in these anti-inflammatory and immune-modulatory strategies (31). Tregs, characterized by the expression of the forkhead transcription factor, FOXP3, and the IL-2 receptor α-chain, CD25, are essential for the prevention of both autoimmunity and excessive inflammatory responses to infection and might also facilitate bacterial colonization processes.

The immunological mechanisms involved in bacterial decolonization are better defined, at least in animal models. Current data indicate that a T helper 17 (Th17) cell-mediated inflammatory response is the key factor responsible for clearing of *S. aureus* from the nostrils (32). Ultimately, the colonization phase appears to trigger an anti-inflammatory response, whereas the decolonization phase triggers a pro-inflammatory response.

## Discussion

The temporal correlation observed herein between bacterial colonization/tic improvement and decolonization/exacerbation was indeed surprising. These findings open interesting new questions regarding the possible biological mechanism(s), and particularly the immunological events, behind both phenomena. During the colonization phase first reported in 2014, anti-streptococcal antibody titers as well as autoantibodies against dopamine receptors D1 and D2 were downregulated and dropped to normal values; the concomitant clinical improvement in motor tic frequency and severity was impressive. During the second colonization phase in 2015, motor tic clinical improvement was also significant and was accompanied by negative autoantibody titers against dopamine receptors and a decrease in anti-streptococcal antibody levels, albeit to a lower extent and with slower kinetics than in the first colonization phase. However, vocal tics were not controlled, because the worst YGTSS score was recorded while *S. aureus* was still present (Figure 1). The reason for the discrepancies is unclear, but we hypothesize that different brain targets with different pathogenetic mechanisms might have accounted for the two tic categories. Moreover, we do not have evidence of any relevant change in the patient lifestyle or of any environmental factor other than infections that could account for the tic severity fluctuations observed.

Whatever the mechanism, the clinical improvement in motor tic severity was temporally correlated with the staphylococcal colonization phases, and possibly also with the decreased anti-dopamine receptor autoantibody levels. These findings are similar to those reported in post-streptococcal Sydenham’s chorea patients, where dopamine D1 and D2 receptor autoantibody levels correlated with symptom severity (33). Our data also support an etiological role for GAS infections in the onset of tics, at least in a subgroup of patients with TS. Our observations, likewise, support the involvement of autoantibodies against dopamine receptors in the pathogenesis of some movement disorders.

This case could not be included in the group of PANDAS because there was neither an evidence for abrupt disease onset, which is required by the inclusion criteria (6), nor a symptom association with GAS isolation (6). In fact, the serological indication of a possible GAS infection could not be confirmed in the current investigation by streptococcal isolation, and the results of microbiological tests for *Streptococcus pyogenes* presence in nasal, throat, and tonsils swabs were always negative. Analyses performed on the parents of the patient revealed a similar intriguing response in the mother: above-normal ASO titers during the 18 months of the 2014–2015 survey, ranging from 222 to 286 IU/mL, with negative swab tests for GAS infection. This reinforces the hypothesis of an underlying genetic immunological imbalance in the daughter (2, 3).

However, Swidsinski and colleagues reported that “various environmental conditions can shield bacteria, rendering them inaccessible for microbial diagnosis through a swab test” (34). We cannot, therefore, exclude in principle a hidden, subclinical, and localized GAS infection that was responsible for maintaining increased anti-streptococcal antibody production.

The lack of an abrupt, dramatic onset of an obsessive–compulsive disorder (OCD), or of a severely restricted food intake, exclude likewise the inclusion of this case in the “broader” category of the pediatric acute-onset neuropsychiatric syndromes (PANS), which include different infectious and non-infectious triggers (35). The case reported herein can be classified in our opinion as a subgroup of TS patients (rare?), where infectious agents could modulate the course of the illness. TS is on the contrary not uncommon (36), being a disorder occurring in up to 1% of children (37–39).

A discrepancy was observed between the decrease of antibody titers against dopamine D1 and D2 receptors and ASO occurring in the colonization phase and the increase of the inflammatory markers during bacterial clearance. The notion presented is that *S. aureus* colonization could trigger an anti-inflammatory modulatory response, whereas the decolonization process may trigger the opposite. An inflammatory rebound response was in fact fairly evident after bacterial clearance (Table 1, period B), given that the ESR, ASTA titer, and WBC counts were all above normal levels during this period compared to the levels occurring before (Table 1, period A) and during the colonization phase (Table 1, period C). Furthermore, CaM kinase II values remained high throughout the entire observation period, suggesting that the “main switch” of the illness was continuously “on,” even during the quiescent state when autoantibodies against dopamine receptors fell to negative values. The rapid reversal of clinical parameters following the clearance of *S. aureus* by the host supports a direct relationship between the decolonization process, the inflammatory response, and tic exacerbation. Transient microinvasions by *S. aureus* could be responsible for the immunological host response, according to the existing models of *S. aureus* nasal carriage (22) and to the increased ASTA titers observed.

Moreover, the shift from an anti-inflammatory modulatory response to a pro-inflammatory state during the clearing process is consistent with the data from animal models showing that a pro-inflammatory, Th17 cell-associated immune response is required for *S. aureus* nasal decolonization, where the *S. aureus* nasal carriage clearance is T cell-dependent and mediated by IL-17A expression and neutrophil influx (32).

Interestingly, human Th17 lymphocytes promote blood–brain barrier (BBB) disruption and central nervous system inflammation during the pathogenesis of multiple sclerosis (MS) (40), another neurological disease in which autoimmunity plays a central role. BBB leakage might be an important step to investigate the pathogenesis of any autoimmune neurological disorder. Also in MS, *S. aureus* is considered a possible risk factor for the clinical exacerbation (41). Future studies on infection-driven immune-inflammatory mechanisms, potentially involved in the pathogenesis of tic disorders, should focus on bacterial virulence factors and immunological host responses that could locally affect BBB permeability allowing autoantibodies to reach their potential brain targets.

## Concluding Remarks

This case is the first demonstration of the modulation of tic manifestation in a *S. aureus* intermittent carrier with TS, during periodically occurring colonization and decolonization phases. A shift occurred from an anti-inflammatory modulatory response during the colonization phase to a pro-inflammatory state during the clearing process. Thus, *S. aureus* nasal carriage possibly provides a new human model for the *in vivo* study of the interplay between infections, immunity, autoimmunity, and tic disorders.

## Author Contributions

EC was responsible for study design and research proposal and contributed to laboratory data interpretation. EG performed data analysis and managed the literature searches. VP performed the neuropsychiatric monitoring and contributed to methodology. All authors contributed to writing the manuscript. All authors read and corrected the manuscript. All authors contributed to and have approved to the final manuscript.

## Conflict of Interest Statement

The authors declare that the research was conducted in the absence of any commercial or financial relationships that could be construed as a potential conflict of interest.

## Abbreviations

ASO, anti-streptolysin O; ASTA, anti-staphylolysin antibody; BBB, blood–brain barrier; CaM kinase II, calcium/calmodulin-dependent protein kinase type II; DNase B, deoxyribonuclease B; ELISA, enzyme-linked immunosorbent assay; ESR, erythrocyte sedimentation rate; GAS, group A streptococcal; MS, multiple sclerosis; PANDAS, pediatric autoimmune neuropsychiatric disorders associated with streptococcal infection; PANS, pediatric acute-onset neuropsychiatric syndromes; OCD, obsessive–compulsive disorder; Th17 cell, T helper 17 cell; WBC, white blood cell; YGTSS, Yale global tic severity scale.
